# In Vitro Antibacterial Activities of Methanolic Extracts of Fruits, Seeds, and Bark of *Zanthoxylum armatum* DC

**DOI:** 10.1155/2020/2803063

**Published:** 2020-06-04

**Authors:** Nirmala Phuyal, Pramod Kumar Jha, Pankaj Prasad Raturi, Sangeeta Rajbhandary

**Affiliations:** ^1^Central Department of Botany, Tribhuvan University, Kirtipur, Kathmandu, Nepal; ^2^Forest Research and Training Center, Ministry of Forests and Environment, Babarmahal, Kathmandu, Nepal; ^3^Ashok Medicinal and Aromatic Plants Center, Dabur Nepal Pvt. Ltd., Janagal, Kavre, Nepal

## Abstract

Crude methanol extracts of fruits, seeds, and bark of *Zanthoxylum armatum* were investigated in vitro for antimicrobial activities against 9 different bacterial strains: *Bacillus subtilis*, *Enterococcus faecalis*, *Proteus vulgaris*, *Pseudomonas aeruginosa*, *Salmonella typhi*, *Shigella dysenteriae*, *Staphylococcus aureus*, methicillin-resistant *Staphylococcus aureus* (MRSA), and *Staphylococcus epidermidis* using agar well diffusion method, and the MBC values were determined. Only 5 bacteria, i.e., *Bacillus subtilis, Enterococcus faecalis,* MRSA, *Staphylococcus aureus*, and *Staphylococcus epidermidis* exhibited antibacterial properties against the different extracts. The fruit and seed extracts showed activities against 5 bacteria, while the bark extract was active against 2 bacteria only (MRSA and *Staphylococcus aureus*). *Staphylococcus aureus* was found to be more susceptible for all the extracts compared to other strains. The maximum ZOI of 20.72 mm was produced by fruits (wild) and 18.10 mm (cultivated) against *Staphylococcus aureus.* Likewise, the least ZOI of 11.73 mm was produced by seeds (wild) and 11.29 mm (cultivated) against *Escherichia faecalis.* Similarly, the lowest MBC value of 0.78 mg/mL was obtained for fruit extracts against MRSA, 1.56 mg/mL for fruits, seeds, and bark extracts against *Bacillus subtilis*, MRSA, and *Staphylococcus aureus*, and highest value of 50 mg/mL for fruits and seeds extracts against *S. epidermidis.* The fruits, seeds, and bark extracts of *Z. armatum* exhibited remarkable antibacterial properties against different pathogenic bacteria causing several diseases, which suggests the potential use of this plant for treating different bacterial diseases such as skin infection, urinary tract infection, dental problems, diarrhea, and dysentery.

## 1. Introduction

Among the 8 species of *Zanthoxylum* (family Rutaceae) found in Nepal, *Zanthoxylum armatum* DC. is one of the most common spice plants with many medicinal values. Commonly known as timur, it is a subdeciduous aromatic shrub or small tree up to 6 m high. It is found in hot valleys of subtropical to temperate Himalayas (Kashmir to Bhutan), northeast India and Pakistan, Laos, Myanmar, Thailand, China, Bangladesh, and Bhutan [1]. In Nepal, it is found from west to east in open places or in forest undergrowth at an altitude of 1000 m to 2500 m [2].

In modern medicine system, a wide range of antimicrobials are used for the treatment of several contagious diseases, which may result in undesirable side effects and serious medical problems [3, 4]. The indiscriminate use of commercial antimicrobials has also resulted in the development of multiple drug resistance. This has urged researchers to identify or extract natural antimicrobials from natural sources with less health severity [5]. Plants are the potential source of novel antimicrobial agents [6, 7]. Herbal medicinal practice, which uses plant sources to cure various infectious diseases, is also getting popularity these days. A number of flavonoids and phenolics, the potential agents for antimicrobial, antioxidant, and anticancer activities, have been identified and isolated from different plants [8, 9].

The leaves, fruits, seeds, and bark of *Z. armatum* possess various medicinal properties and have been used traditionally in several diseases as carminative, antipyretic, appetizer, stomachic, dyspepsia, and in toothache [2, 10–12]. A wide array of chemical compounds such as alkaloids, flavonoids, glycosides, terpenoids, steroids, phenols, lignins, coumarins, and benzoids [13–18] have been reported from different parts of the plant, which are responsible for several biological activities including antimicrobial, antioxidant, antipyretic, larvicidal, and anti-inflammatory properties. Young shoots are used as toothbrush and useful for curing gum diseases and toothache. The main components of the essential oil are linalool and limonene. The fruits and seeds are employed as an aromatic tonic in fever, dyspepsia, and expelling roundworms [2, 11]. The fruits are used as condiments and spices. Several indigenous medicinal practices of *Z. armatum* in various ailments indicate that the plant may have constituents of antimicrobial potential. Hence, an attempt has been made in this study to evaluate the potential antimicrobial activities of methanolic extracts of fruits, seeds, and bark of *Zanthoxylum armatum* collected from wild and cultivated populations in Nepal.

## 2. Materials and Methods

### 2.1. Collection and Processing of Samples

The fresh fruits, seeds, and bark of *Z. armatum* were collected from wild and cultivated populations from Salyan District of Nepal during May 2018. The samples were cleaned and shade dried for a week before the extraction procedure. The herbarium of the voucher specimens was prepared, which are deposited at the National Herbarium and Plant Laboratories (KATH) NPZA 20-NPZA 50.

### 2.2. Extraction of the Samples

The dried samples were then powdered separately in a grinder. Known weight of the powdered samples was loaded in a thimble and put inside the Soxhlet apparatus. They were then successively extracted by the hot Soxhlet extraction method. The apparatus was run for 72 hours till the colored solvent appeared in the siphon for obtaining the crude extracts of the samples. After complete extraction, the solvents were evaporated in a rotary vacuum evaporator at 65°C under reduced pressure. The obtained extracts were then dried in water bath. The dried extracts were sealed inside 20 mL sterilized culture tubes and stored in a refrigerator at 2–8°C for further analysis [19].

### 2.3. Antibacterial Activity

Methanolic extracts of the fruits, seeds, and bark of *Zanthoxylum armatum* from wild and cultivated populations were screened against different bacteria by agar well diffusion methods as described by Perez et al., 1990 [20].

#### 2.3.1. Test Organisms

The extracts were screened against 9 different bacterial strains: *Bacillus subtilis* ATCC 6051, *Enterococcus faecalis* ATCC 29212, *Proteus vulgaris* ATCC 6380, *Pseudomonas aeruginosa* ATCC 9027, *Salmonella typhi* clinical sample, *Shigella dysenteriae* clinical sample, *Staphylococcus aures* ATCC 6538P, methicillin-resistant *Staphylococcus aureus* (MRSA) clinical sample, and *Staphylococcus epidermidis* ATCC 1228. Chloramphenicol was used as the standard antibacterial agent.

#### 2.3.2. Culture of the Bacteria

Nutrient agar (NA) media were used for culturing the bacteria. Required amount of the media was prepared, autoclaved, then cooled to 40°C and poured to sterilized Petri dishes, and allowed to solidify. Required numbers of colonies of test organisms were cultured in the respective plates and kept inside an incubator for 18–24 hours at 35°C prior to inoculation. All these experiments were carried out aseptically in a biosafety cabinet.

#### 2.3.3. Preparation of Inoculum/Suspension

Required colonies (1.5 × 10^8^ cfu·mL^−1^) of freshly cultured bacteria were inoculated aseptically to glass vials containing normal saline. The suspension was homogenized by vortexing the solution and compared with the turbidity of 0.5 McFarland standard turbidity recommended by the WHO, 1991 [21], for the antimicrobial susceptibility test.

#### 2.3.4. Screening and Evaluation of Zone of Inhibition (ZOI)

Carpet culture of the prepared cell suspensions was done by uniformly spreading the suspensions with the help of cotton swabs over the dry surface of Mueller-Hinton Agar (MHA) plates. These processes were repeated thrice rotating the plate through an angle of 60°C between each streaking, and the inoculated plates were left for maximum 15 minutes to allow absorption of excess surface moisture.

Four wells, each of 6 mm diameter, were bored in the inoculated plates using a sterile cork borer. 50 *μ*L of the 20% test solution of the extracts dissolved in methanol, positive control, and negative control was poured into the respective wells. Chloramphenicol and methanol were used as positive and negative controls, respectively. Thus inoculated plates were put inside the incubator at 35 ± 2°C. After 18–24 h, the plates were evaluated for the zone of inhibition (ZOI). The diameter of each ZOI was measured in millimeters by digital Vernier Caliper.

#### 2.3.5. Determination of Minimum Inhibitory Concentration (MIC) and Minimum Bactericidal Concentration (MBC)

Minimum inhibitory concentration (MIC) was determined by observing the visible growth of the test organisms in two-fold serial-diluted antibacterial substances in nutrient broth (NB) culture media while MBC was determined by subculturing the MIC cultures on suitable agar plates [22].

*(1). Minimum Inhibitory Concentration (MIC)*. A set of 12 sterilized labelled vials containing 1 mL of presterilized Mueller-Hinton Broth (MHB) were prepared. 1 mL of the respective extract solution was added to 11 vials only, and the first vial was used as negative growth control. After complete homogenization, 1 mL of the solution containing nutrient broth and extract solution was transferred to the second vial containing 1 mL of nutrient broth. In the same manner, two-fold serial dilution was prepared up to the 10th vial. Thereafter, 1 mL of content was discarded from the 10th vial and the 11th vial was used as positive control. Now, all the vials except the 1st and last contain equal volume, i.e., 1 mL, gradually decreasing concentration of the solution. To all these vials, 20 *μ*L of bacterial suspension (turbidity equal to a 0.5 McFarland standard, supposed to have 1.5 × 106 CFU/mL) was put into each vial and mixed thoroughly. The vials were then incubated at 37°C for 24 h. MIC was taken as the lowest concentration that prevented the growth of bacterial culture [23].

Minimum inhibitory concentration (MIC) is defined as the lowest concentration of an antimicrobial agent that prevents the visible growth of organisms as detected by lack of visible turbidity. The clarity of the solution (absence of turbidity) indicates the inhibition of microbes. However, whether the turbidity was due to the growth of the bacteria or due to the turbidity of the plant extract itself was difficult to determine. Hence, MBC was determined to find out the minimum concentration of the extract that kills the microorganisms.

*(2). Minimal Bactericidal Concentration (MBC)*. Minimum bactericidal concentration (MBC) is the lowest concentration of an antimicrobial agent required to kill the microorganism. The MBC values were determined by subculturing all the test dilutions of the extracts on the fresh nutrient agar (NA) medium and incubating further for 24 hours at 37°C. The lowest concentration of the extract (mg/mL) that did not result in the appearance of a single bacterial colony on the solid medium was regarded as the MBC [24].

## 3. Results and Discussion

Methanolic extracts of fruits, seeds, and bark of *Z. armatum* were tested against various bacteria. Antibacterial properties of the extracts were compared with chloramphenicol as positive control. The diameter of zone of inhibition (ZOI) produced by the extracts on the particular microorganisms was measured in mm for the estimation of their antibacterial activities. Amongst the organisms tested for antibacterial properties, fruit and seed extracts showed antibacterial activity against *Bacillus subtilis, Escherichia faecalis*, MRSA, *Staphylococcus aureus*, and *Staphylococcus epidermidis* and the bark extract against MRSA and *Staphylococcus aureus* only, while the extracts did not produce any ZOI for the rest of the organisms.

**Figure 1:**
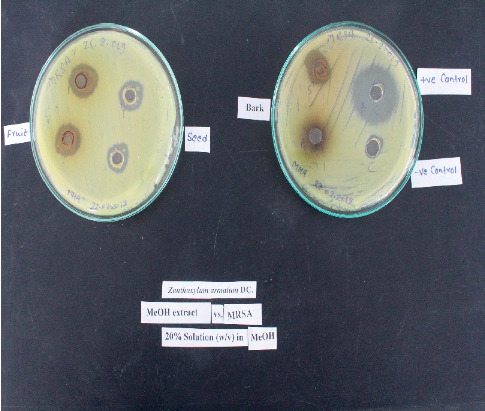
ZOI of different extracts against MRSA.

**Figure 2:**
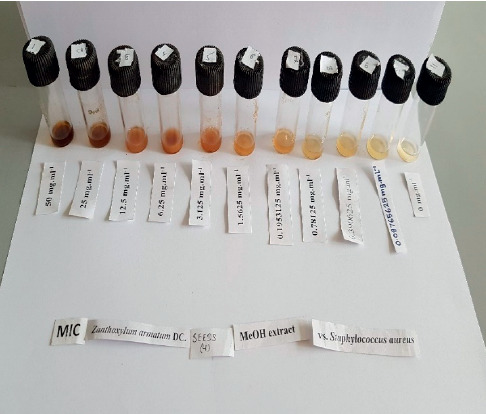
MIC of seeds (cultivated) against *Staphylococcus aureus*.

**Figure 3:**
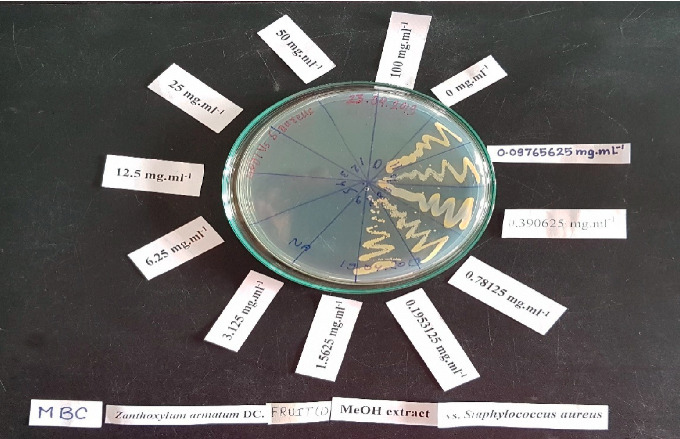
MBC determination of fruits (wild) against *Staphylococcus aureus*.

Antibacterial properties of the fruits, seeds, and bark extracts against different bacterial strains and the ZOI are presented in Table 1 and Figures 1–3. The most sensitive strain was *Staphylococcus aureus* for all the fruits, seeds, and bark extracts as they showed maximum ZOI for *S. aureus* compared to other strains. The highest ZOI was produced by the extracts against *S. aureus*, 20.72 mm for wild fruits and 18.10 mm for cultivated fruits, 17.83 mm for wild seeds and 16.33 mm for cultivated, and the ZOI for bark was 17.01 mm for wild and 16.44 mm for cultivated. The least activity was shown by the seed extracts against *Escherichia faecalis,* with a minimum ZOI of 11.73 mm (wild) and 11.29 mm (cultivated).

The fruits extract showed highest inhibition against the bacterial strains than seed and bark extracts. The ZOI of fruits against *B. subtilis* was 16.24 mm (wild) and 17.04 (cultivated); *Escherichia faecalis* 14.28 mm (wild) and 14.62 mm (cultivated); MRSA 15.02 mm (wild) and 16.28 mm (cultivated); and *Staphylococcus epidermidis* 16.38 mm (wild) and 16.19 mm (cultivated). The seed extracts showed moderate activities against the tested organisms. The ZOI of seeds for *Bacillus subtilis* was 15.72 mm (wild) and 16.28 mm (cultivated); MRSA 17.79 mm (wild) and 16.44 mm (cultivated); and *Staphylococcus epidermidis* 15.58 mm (wild) and 13.25 mm (cultivated). The ZOI of bark extract against MRSA was 14.30 mm (wild) and 13.28 mm (cultivated), and that for *Staphylococcus aureus* was 17.02 mm (wild) and 16.44 (cultivated) (Table 1).

**Figure 4:**
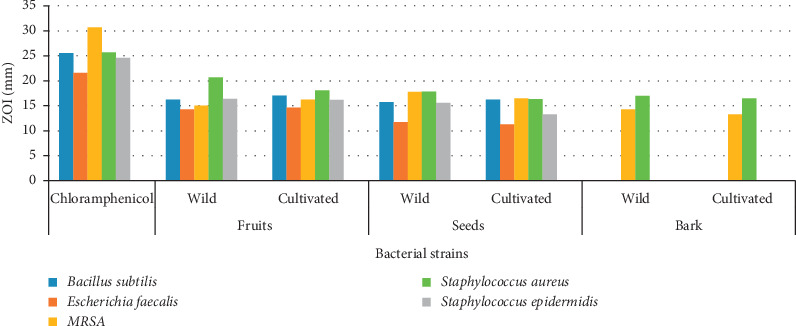
ZOI produced by the plant extracts against different bacteria.

The antibacterial activities of different extracts showed variable results for wild and cultivated populations and were found to be independent of the habitat factors. Some of the extracts from wild populations showed good antibacterial properties, and some of the cultivated samples showed better results. All the extracts of fruits, seeds, and bark were found to be less effective than the standard antibiotic used in the present study (Figure 4).

**Figure 5:**
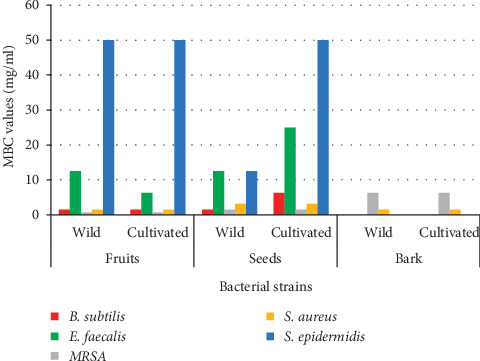
MBC values of the extracts against different bacteria.

The minimum bactericidal concentration (MBC) values ranged from 0.78 mg/mL to 50 mg/mL. The lowest MBC value of 0.78 mg/mL was exhibited by fruit extracts against MRSA and the highest, i.e., 50 mg/mL, was by seeds (wild) and fruits for *S. epidermidis.* The results are presented in Table 2. The MBC value of 1.56 mg/mL was obtained for fruits (wild and cultivated) and seeds (wild) against *B. subtilis;* seeds (wild and cultivated) against MRSA; and fruits (wild and cultivated) and bark (wild and cultivated) against *S. aureus*. Similarly, the MBC value was 3.12 mg/mL for seeds (wild and cultivated) against *S. aureus*. The MBC values of the extracts against *E. faecalis* and *S. epidermidis* were comparatively higher (Figure 5).

Several experiments have demonstrated considerable amount of antibacterial activities of *Z. armatum* leaf, fruit, seed, and bark extracts against different bacterial strains [25–30]. The ZOI produced by the ethanolic and hexane extracts of barks of *Z. armatum* showed antibacterial activities against different bacteria. The ZOI produced by the extract against *B. subtilis* and *E. coli* both were 11.67 mm and against *S. aureus* was 17.33 mm for ethanolic extract and 17 mm for hexane extract [26]. Similarly, in another study, the methanolic extracts of the fruits produced 7 mm ZOI against *S. aureus*, and the MBC value was 2.5 mg/mL and 23 mm ZOI and MBC value >10 for *B. subtilis* [30]. The methanolic extract of bark showed 28.7 mm ZOI against *S. aureus* [31].

The antimicrobial activities exhibited by the different plant extracts against a particular organism depend upon several extrinsic and intrinsic factors. The diffusion ability of agar media may cause the extract to produce less ZOI than its actual efficacy. Hence, the MBC value was determined to actually find out the minimum concentration of the extracts required inhibits the growth of the test organisms [32].

Flavonoids and other phenolics exhibit a wide range of fascinating biological activities such as antimicrobial, antiviral, antioxidant, and anticancer properties [33]. The antibacterial activities of fruit extracts of *Z. armatum* were comparatively better than seed and bark extracts. It might be due to the higher phenolic and flavonoid contents in fruits than seeds and bark [18]. The antibacterial properties of the crude extracts may be attributed to the collegial effects of several phytoconstituents present in the plant. *Z. armatum* has been reported to produce structurally diverse chemicals including terpenoids, flavonoids, coumarins, sterols, and alkaloids that show antibacterial activity. Many active components have been identified from the plant that might be developed into novel drugs. Therefore, further emphasis should be on screening, isolation, and characterization of the individual components responsible for different antibacterial activities and their underlying mechanism of action. However, additional studies are required to quantify the acute and chronic toxicity in animals before clinical trials [12, 34].

The tested extracts have potential antibacterial activities against different pathogens causing several infectious diseases in humans. These experiments partially validate the use of this plant in several traditional medicinal practices to cure various diseases. Hence, further research should be focused towards exploiting the possible uses of *Zanthoxylum armatum* for treating different bacterial diseases such as urinary tract infection, skin infection, diarrhea, dysentery, and tooth problems (Figures 1–3).

## 4. Conclusions

The results of the study revealed that the crude extracts of fruits, seeds, and bark of *Zanthoxylum armatum* from different habitats have antibacterial properties against several infectious pathogens causing several diseases in humans. The fruits were found to be more active against the bacteria than seeds or bark, and the fruits showed highest ZOI against *Staphylococcus aureus*. Similarly, the seeds were also found to have good antibacterial properties. Bark exhibited activities against MRSA and *Staphylococcus aureus* only. The findings of this study support the traditional usage of the plants in herbal medicinal practices. Medicinal plants contain a variety of compounds that can be developed into several drugs for the safe usage and benefit to mankind. Since the indiscriminate use of modern antibiotics may cause severe health hazards, these plants can be safe alternatives to treat several diseases. The antibacterial properties as exhibited by the fruits, seeds, and bark of *Z. armatum* suggest the potential use of this plant in skin infection, urinary tract infection, dental problems, diarrhea, and dysentery. Hence, further research should be directed towards the extensive in vivo and clinical studies along with the mechanism of action of the antibacterial activities, which justifies the rational for their traditional uses and also leads to the development of novel plant-based antimicrobials for safe health care services.

## Figures and Tables

**Table 1:** ZOI of methanolic extracts of *Zanthoxylum armatum* fruits, seeds, and bark.

S. No.	Name of the organisms	Chloramphenicol	Zone of inhibition (ZOI (mm))					
			Fruits		Seeds		Bark	
			Wild	Cultivated	Wild	Cultivated	Wild	Cultivated
1	*Bacillus subtilis*	25.52	16.24	17.04	15.72	16.28	0	0
2	*Escherichia faecalis*	21.61	14.28	14.62	11.73	11.29	0	0
3	MRSA	30.70	15.02	16.28	17.79	16.44	14.30	13.28
4	*Staphylococcus aureus*	25.64	20.72	18.10	17.83	16.33	17.01	16.44
5	*Staphylococcus epidermidis*	24.55	16.38	16.19	15.58	13.25	0	0
6	*Proteus vulgaris*	0	0	0	0	0	0	0
7	*Pseudomonas aeruginosa*	0	0	0	0	0	0	0
8	*Salmonella typhi*	0	0	0	0	0	0	0
9	*Shigella dysenteriae*	0	0	0	0	0	0	0

**Table 2:** MBC values of the extracts against different bacteria.

S. No.	Organisms	MBC values (mg/mL)					
		Fruits		Seeds		Bark	
		Wild	Cultivated	Wild	Cultivated	Wild	Cultivated
1	*B. subtilis*	1.56	1.56	1.56	6.25	0	0
2	*E. faecalis*	12.5	6.25	12.5	25	0	0
3	MRSA	0.78	0.78	1.56	1.56	6.25	6.25
4	*S. aureus*	1.56	1.56	3.12	3.12	1.56	1.56
5	*S. epidermidis*	50	50	12.5	50	0	0

## Data Availability

The data used to support the findings of this study are included within the article.
